# Hepatitis B Virus Genotype C is Predominant in Myanmar

**DOI:** 10.3390/diseases6010003

**Published:** 2017-12-26

**Authors:** Nan Nwe Win, Shingo Nakamoto, Myint Myint Sein, Mitsuhiko Moriyama, Tatsuo Kanda, Hiroshi Shirasawa

**Affiliations:** 1 Department of Molecular Virology, Graduate School of Medicine, Chiba University, Inohana 1-8-1, Chuo-ku, Chiba 260-8677, Japan; nannwewin@gmail.com (N.N.W.); nakamotoer@yahoo.co.jp (S.N.); mmseindr@gmail.com (M.M.S.); sirasawa@faculty.chiba-u.jp (H.S.); 2 Division of Gastroenterology and Hepatology, Department of Medicine, Nihon University School of Medicine, 30-1 Oyaguchi-Kamicho, Itabashi-ku, Tokyo 173-8610, Japan; moriyama.mitsuhiko@nihon-u.ac.jp

**Keywords:** HBV genotypes, hepatitis B virus, Myanmar

## Abstract

Myanmar is adjacent to India, Bangladesh, Thailand, Laos and China. In Myanmar, the prevalence of hepatitis B virus (HBV) infections is 6.5% and accounts for 60% of hepatocellular carcinoma. HBV has nine genotypes that have been identified by molecular genetic analysis. HBV genotypes are associated with several clinical features. We reviewed the prevalence of HBV genotypes in Myanmar and neighboring countries. We also reviewed HBV genotypes in refugees from Myanmar. HBV subgenotype C1 is predominant in Myanmar. As HBV genotype C is associated with hepatocellular carcinoma (HCC), it is important to screen for cirrhosis and HCC and to prevent their development in HBV-infected individuals of Myanmar.

## 1. Introduction

Hepatitis B virus (HBV) infection is one of the major causes of chronic hepatitis and cirrhosis and occasionally leads to hepatocellular carcinoma (HCC) [1]. HBV, a member of the family Hepadnaviridae, is a partially double-stranded DNA virus of approximately 3200 bp in length, with four overlapping open-reading frames: *pre-C/C*, which encodes for e antigen (HBeAg) and core protein (HBcAg); *P* for polymerase (reverse transcriptase); *S* for surface proteins [three forms of HBsAg: small (S), middle (M) and large (L)]; and *X* for a transcriptional transactivator protein [2]. HBV has been classified phylogenetically into 9 genotypes, A-I [3]. HBV genotypes C and D are associated with the occurrence of HCC. HBV genotype A is associated with chronicity after acute hepatitis B. Diversity of the HBV genome coding for HBsAg accounts for the four serological subtypes: adw, adr, ayw and ayr [4].

## 2. HBV Genotypes in Neighboring Countries of Myanmar

Myanmar is the northwestern-most country on the mainland of Southeast Asia and is bordered by Bangladesh, India, China, Laos and Thailand. Although HBV genotypes in neighboring countries seem to have an impact on HBV genotypes in Myanmar, HBV genotype C is not the predominant genotype in India, Bangladesh and southern Chinese provinces (Table 1).

In Arunachal Pradesh which is located in northeast India, the predominant HBV genotype is genotype A (41.6%), followed by genotypes C (27.8%) and D (11.1%) [5]. Among healthy blood donors in the eastern part of North India, the most common HBV genotypes were A (54.0%), D (21.3%), C (10.0%), B (6.7%), E (6.0%) and F (2.0%) [6]. In Kolkata, in eastern India, HBV genotypes D, A and C have prevalence rates of 57.0%, 25.0% and 18.0%, respectively among HBV carriers [7] (Table 1). 

In Bangladesh, 48.7%, 28.2% and 23.1% of chronic HBV patients have HBV genotypes C, D and A, respectively. The predominant subtypes in Bangladesh was adr (41%), followed by subtypes adw2 (28.2%), ayw3 (25.6%) and others [8]. Rhaman et al. [8] collected sera from 50 chronic hepatitis B patients who attended one of the largest hospitals in the Dhaka city, which provides services to patients coming from different parts of the country. HBV genotypes were determined by HBV genotype specific primers and direct-sequencing of *pre-S1/pre-S2/S* region of HBV (Table 1).

However, Shaha et al. [9] reported that in Bangladesh, genotype D is the predominant genotype (73.7%), with subtypes ayw3 (64.3%) and ayw2 (35.7%); followed by genotype A, with subtype adw2 (15.8%); and genotype C, with subtype adr (10.5%). They collected and studied sera from 385 patients with jaundice like illness from different medical college hospitals of Dhaka city [9]. HBV genotypes were determined by direct-sequencing of partial *S-gene* of HBV (Table 1).

The most common HBV genotypes among hepatitis B carriers in Thailand were genotype C (72.0%), genotype B (25.2%) and genotype D (2.8%) [10]. Louisirirotchanakul et al. [11] found that HBV genotype C (87.2%) and genotype B (10.4%) were the most prevalent in HBsAg positive donors in Thailand. Surprisingly, in that study, one patient was infected with a genotype H strain (1.2%), while another patient was infected with a genotype G strain (1.2%) (Table 1).

In Northern provinces of China, the prevalence of the two main HBV genotypes B and C, respectively, are 18% and 81% in Beijing; 12% and 31% in Xinjiang; 11% and 75% in Gansu; and 0% and 99% in Shandong. In contrast, in Southern provinces, the prevalence of HBV genotype B is higher than that of genotype C, respectively: at 81% and 18% in Hunan; at 62% and 32% in Fujian; at 49% and 46% in Yunnan; and at 53% and 46% in Guangdong. Therefore, HBV genotype C is predominant in northern China while genotype B is more common in southern China [12]. This study included 1143 patients with chronic HBV infection from 9 hospitals in China [12]. HBV genotypes were determined by polymerase chain reaction (PCR)-restriction fragment length polymorphism (RFLP) using an amplicon of *HBV surface gene* (Table 1).

Yunnan is the most southwestern province in China and it shares a border with Myanmar in the west, Laos in the south and Vietnam in the southeast. In Yunnan Province, genotype C was the most prevalent, infecting 62.5% of patients, followed by genotype B (33.3%), genotype I (2.78%) and C/D recombinants (1.39%). HBV genotype B consists of a single subgenotype, B2, while HBV genotype C can be classified into several subgenotypes (C1, C2, C5 and C7). Clusters of subgenotypes B2 and C2 consists of strains from China and other East Asian countries, while subgenotypes C1, C5 and C7 and genotype I form a cluster together with strains from Southeast Asia [13]. This study included 80 HBV-positive serum samples, collected from hepatitis patients who sought medical service at the First People’s Hospital of Yunnan Province from 2011 to 2012 [13]. HBV genotypes and subtypes were determined by phylogenetic analysis of pre-S/S sequences of HBV strains (Table 1).

Similarly, Kang et al. [14] reported that HBV genotype C was the most prevalent genotype (76.9%) in Yunnan, followed by genotypes B (15.4%), D (5.1%) and I (2.5%). They collected 39 HBsAg-positive sera from people who had a physical examination. HBV genotypes were determined by nested PCR and sequencing of *HBV surface genes* [14]. In addition, HBV genotypes C, B, D, mixed genotype B/C and mixed genotype A/C were found in 54.8%, 38.1%, 0.8% 1.6% and 1.6%, respectively, of patients with chronic HBV infections from Yunnan Province [15]. They included 126 HBV patients with chronic various diseases. HBV genotypes were determined reverse dot blot hybridization [15].

However, Shen et al. [16] found that the genotype prevalence was 59.6% for B, 21.1% for C and 19.3% for B/C, with subgenotypes Ba, Cs and Ce seen in asymptomatic local people of Yunnan. They examined sera from 147 HBsAg-positive asymptomatic people from ethnic groups in West Yunnan. HBV genotypes were determined by genotype-specific PCR method [16] (Table 1).

## 3. HBV Genotypes of Myanmar

Hepatitis virus infection are widespread in Myanmar and patients with acute and chronic hepatitis are often observed [17]. It is necessary to know the prevalence of hepatitis virus as well as their genotypes such as HBV genotypes. HBV genotype C (77.0%) was the most prevalent, followed by genotype A (8.0%), among 16 healthy individuals and 48 patients with liver diseases who were examined at the Yangon General Hospital, Yangon, Myanmar (Table 1) [17].

Among migrant workers traveling from Myanmar to Thailand, the most prevalent HBV genotype was genotype C (97.5%), followed by genotype B (1.25%) and genotype D (1.25%). For antigenic subtype distribution, adr was the most common (71.25%), followed by ayw (2.5%) and adw (1.25%) [18]. Huy et al. [19] also investigated the prevalence of the HBV variant with the *pre-S mutation* in different geographic regions. This mutation was the most prevalent in Vietnam (36%), followed by Nepal (27.3%), Myanmar (23.3%), China (22.4%), Korea (14.3%), Thailand (10.5%), Japan (7.7%) and Ghana (4.3%) (Table 1). 

Using phylogenetic analysis of the *pre-S1*/*pre-S2* genes from isolates in Asia, Huy et al. [20] reported that HBV C1 was found only in Southeast Asia, including Vietnam, Myanmar and Thailand, while HBV C2 was found in Far East Asia, including Japan, Korea and China. Zaw et al. [21] reported that the sero-prevalence of HBsAg was 8.7% among patients co-infected with HBV and HIV in Myanmar.

## 4. HBV Genotypes of Immigrants from Myanmar

To elucidate HBV genotypes in Myanmar, we also searched publications and examined HBV genotypes of immigrants from Myanmar. Immigration from countries with high HBV endemicity to those with low HBV endemicity warrants particular attention in order to prevent the spread of HBV infection to the native population [22]. More than 50% of Myanmar refugees bound for United States tested positive for anti-HBs [23]. The HBV DNA detection rate was 18.9% and the median HBV viral load was 1.32 × 10^4^ IU/mL in United States-bound refugees from Myanmar. The HBV genotype C was predominant and genotype G was found in a single sample but HBV genotype A was not found in United States-bound refugees from Myanmar [23]. Schulz et al. [24] characterized 76 adult immigrants from Myanmar in Australia: and found that 8 (10.5%) and 68 (89.5%) had HBV genotypes B and C, respectively. HBV subgenotype C1 was found in 67/68 patients (98.5%).

## 5. Conclusions

The results of previous studies indicate that HBV subgenotype C1 seems to be predominant in Myanmar. Several studies revealed that diversity within the *HBV genome*, such as genotype C, is directly associated with cirrhosis and with HCC incidence and outcomes [25,26,27,28,29,30]. It may be important to screen for cirrhosis and HCC as well as HBV vaccination and to prevent them from developing with proper treatments. As the size of the groups analyzed for HBV genotypes is very small (Table 1), further studies should be needed at this point.

## Figures and Tables

**Table 1:** Number of patients with various HBV genotypes (GTs) in Myanmar and neighboring countries.

Authors [Ref.]	Area	Total	GT-A	GT-B	GT-C	GT-D	Others
Borkakoty et al. [5]	India	29	12	-	8	3	6
Kumar et al. [6]	India	150	81	10	15	32	12
Banerjee et al. [7]	India	230	57	-	42	131	-
Rahman et al. [8]	Bangladesh	39	9	-	19	11	-
Shaha et al. [9]	Bangladesh	19	3	-	2	14	-
Sugauchi et al. [10]	Thailand	107	-	27	77	3	-
Louisirirotchanakul et al. [11]	Thailand	86	-	9	75	-	2
Zeng et al. [12]	Yunnan, China	85	1	42	39	-	3
Wang et al. [13]	Yunnan, China	72	-	24	45	1	2
Kang et al. [14]	Yunnan, China	39	-	6	30	2	1
You et al. [15]	Yunnan, China	126	-	48	69	1	8
Shen et al. [16]	Yunnan, China	147	-	34	12	-	11
Nakai et al. [17]	Yangon, Myanmar	64	5	-	49	-	10
Sa-Nguanmoo et al. [18]	Immigrant from Myanmar	80	-	1	78	1	-
Huy et al. [19]	Myanmar	30	-	-	30	-	-

- not detected; Others include undetermined, mixed GTs and other GTs.
